# Surgical outcomes in patients with lamellar macular holes selected based on the optical coherence tomography consensus definition

**DOI:** 10.1186/s40942-021-00297-6

**Published:** 2021-04-13

**Authors:** Ismael Chehaibou, Elise Philippakis, Valérie Mané, Carlo Lavia, Aude Couturier, Alain Gaudric, Ramin Tadayoni

**Affiliations:** 1 Ophthalmology Department, AP-HP, Hôpital Lariboisière, Université de Paris, 2 rue Ambroise Paré, 75010 Paris, France; 2 Surgical Department, Ophthalmology Service, Azienda Sanitaria Locale TO 5, 10023 Chieri, Italy

**Keywords:** Epiretinal membrane (ERM), Lamellar hole-associated epiretinal proliferation (LHEP), Lamellar macular hole (LMH), Macular pseudohole (MPH), Spectral-domain optical coherence tomography (SD-OCT), Vitrectomy

## Abstract

**Purpose:**

The surgical indication for lamellar macular holes (LMH) is controversial due to a misclassification of different macular diseases. A consensus based on an optical coherence tomography (OCT) definition has recently been suggested. The aim of this study was to investigate the surgical outcomes of patients with LMH selected based on this OCT-based consensus definition.

**Methods:**

Retrospective review of patients who underwent surgery for LMH with a follow-up of at least 3 months. Anatomical OCT criteria for the diagnosis of LMH were the presence of an irregular foveal contour with foveal cavitation and a loss of retinal tissue. Cases of macular pseudoholes and epiretinal membrane foveoschisis were excluded. Surgery consisted in pars plana vitrectomy with centripetal peri-hole peeling of epiretinal proliferation and internal limiting membrane. Pre- and postoperative visual acuities (VA) were compared, and changes in OCT anatomical features, including the restoration of the foveal profile and outer retinal layers, were assessed.

**Results:**

Eleven eyes of 11 patients were included, of which 9 eyes (81.8%) showed proliferation on preoperative OCT. The mean VA improved from 0.44 ± 0.19 LogMAR (20/55 Snellen equivalent) to 0.16 ± 0.08 LogMAR (20/28 Snellen equivalent), after a mean follow-up of 7.2 ± 2.9 months (*P* = 0.02). Postoperatively, all eyes showed a restored foveal profile. The mean central foveal thickness increased from 127.6 ± 29.9 μm to 209.0 ± 44.0 μm (*P *= 0.001). At baseline, ellipsoid zone disruption and external limiting membrane disruption were found in 9 and 7 eyes, respectively. Postoperatively, the ellipsoid zone and external limiting membrane were restored in respectively 6/9 eyes (66.7%) and 5/7 eyes (71.4%). No cases of postoperative full-thickness macular hole were found.

**Conclusion:**

In patients with LMH carefully selected based on the recent OCT-based criteria and showing a loss of retinal tissue, the foveal architecture was restored and the VA was improved after vitrectomy with peri-hole peeling for epiretinal proliferation.

**Supplementary information:**

The online version contains supplementary material available at 10.1186/s40942-021-00297-6.

## Introduction

Lamellar macular hole (LMH) is a retinal condition characterized by a partial-thickness foveal defect, originally described using biomicroscopy by Gass in 1975. With the advances in retinal imaging and the emergence of the optical coherence tomography (OCT) technology, our ability to detect subtle foveal profile lesions has improved and many different macular conditions have been referred to as LMH [1]. As a consequence, the conclusions of previous reports on LMH surgery have been limited by the lack of consensus on LMH definition. The senior author of the present paper (RT) has recently participated in an international panel of vitreoretinal experts to clarify the OCT definition of LMH [2]. The main diagnostic criteria of LMH include the presence of an irregular foveal contour, the presence of a foveal cavity with undermined edges and an apparent loss of retinal tissue [2]. The minor diagnostic criteria are the presence of epiretinal proliferation, the presence of a foveal bump and a disruption of the ellipsoid zone (EZ) [2]. This consensus definition aimed to distinguish LMH from other related tractional macular diseases such as macular pseudoholes and epiretinal membrane (ERM) foveoschisis, which were previously also called “tractional lamellar macular holes”.

While conventional ERM macular peeling allows restoring the anatomy and improving the vision of patients with macular pseudoholes and ERM foveoschisis [3, 4], “true” LMH have been considered a degenerative condition, stable over time, for which surgery was generally not offered and clinical observation was the rule [5, 6]. However, recent long-term studies of their natural course have shown that LMH eyes may progressively lose retinal tissue, experience a disruption of the outer retinal layers, and a subsequent decrease in visual acuity (VA) [7]. Hence, a few studies have focused on the surgical management of LMH and reported that the retinal defect may be closed by macular peeling, with a potential functional improvement [8–10]. However, other authors have reported negative surgical outcomes with a concern about the risk of postoperative full-thickness macular hole (FTMH) and there is to date no consensus on LMH surgery [6, 11, 12]. In the absence of a precise OCT definition of LMH and due to the inclusion of various distinct clinical entities such as macular pseudoholes and ERM foveoschisis, the conclusions of previous LMH surgical reports could be wrong. The recent OCT-based consensus definition of LMH allows clinicians to use a homogeneous language and valid interstudy comparisons.

Thus, the aim of this study was to investigate the functional and anatomical outcomes of patients with a diagnosis of LMH as defined by the recent international consensus, and treated with a cautious surgical approach limiting the traction on the residual foveal tissue.

## Methods

### Design

In this retrospective study of consecutive cases, the medical records and SD-OCT scans of patients who underwent pars plana vitrectomy (PPV) for LMH in the department of Ophthalmology of Lariboisière hospital, Paris, France, from January 2016 to December 2018, were reviewed. Before 2016, surgery for LMH was rarely performed, and only one patient operated in 2011 was retrieved and also included. This study adhered to the principles of the Declaration of Helsinki and was approved by the ethics committee of the French Society of Ophthalmology (IRB 00008855 Société Française d’Ophtalmologie IRB#1).

### Population

Inclusion criteria were patients with LMH, treated with PPV, with a follow-up of at least 3 months, and who underwent preoperative and postoperative spectral-domain optical coherence tomography (SD-OCT).

Anatomical SD-OCT criteria for the diagnosis of LMH included an irregular foveal contour with foveal cavitation and an apparent loss of retinal tissue [2]. Epiretinal proliferation, also called “lamellar hole-associated epiretinal proliferation” (LHEP), was defined as a thick and homogeneous material with medium reflectivity, and no associated retinal contraction. The absence of contraction is visible, at best, on en face OCT. The presence of epiretinal proliferation was not a criterion required for the diagnosis of LMH [2, 13]. In contrast, ERM was defined as a thin highly reflective line visible on the macular surface resulting in retinal folds on the B-scan or en face OCT. Intraretinal cavitation was defined as hyporeflective cavities in any retinal layer [14].

Exclusion criteria were the presence of a macular pseudohole, defined by the presence of a fovea-sparing ERM, a steepened foveal profile and an increased macular thickness, or ERM foveoschisis, defined by the presence of an ERM and a schisis at the Henle’s fiber layer [2, 15]. Eyes with chronic cystoid macular edema regardless of its cause, advanced age-related macular degeneration, high myopia (> 6 diopters) and advanced glaucoma were also excluded.

### Clinical examination

Comprehensive ophthalmologic examinations included corrected distance VA, corrected near VA, the presence of metamorphopsia, slit-lamp biomicroscopy and fundus examination. Distance VA was measured on a Snellen chart and converted into logarithm of the minimal angle of resolution (LogMAR) values for statistical purposes, while near VA was measured on a Parinaud chart. The presence of metamorphopsia was evaluated using the Amsler grid.

### OCT image acquisition

In all cases, eye-tracked SD-OCT images were obtained using the Cirrus SD-OCT 5000 device (Carl Zeiss Meditec, Humphrey division, Dublin, California, USA) before and 3 months after surgery and at the last follow-up visit. Image acquisition included a macular cube of 128 × 512 (20 × 20°, 6 × 6 mm, spacing of 47 µm), and two vertical and horizontal high-definition 5-line raster scans centered on the fovea spaced 75 µm apart. From the macular cube acquisition, the advanced mode visualization provided a 4 x 4 mm en face image of the inner retinal surface. Additionally, some patients were also imaged with the Spectralis SD-OCT device (Heidelberg, Engineering GmbH, Heidelberg, Germany) and a high-resolution B-scan macular volume (5° × 20°, 29 lines, 6 mm, spacing of 60 mm, ART 5, high resolution, 768 × 496) and two high-resolution cross-scans of 30° horizontally and vertically (9 mm, ART 5, 1536 × 496) were obtained.

All preoperative SD-OCT scans were reviewed independently by at least two retina specialists (IC, VM and EP) to confirm the diagnosis of LMH. In case of concomitant extrafoveal ERM, OCT B-scan criteria and en face OCT images allowed differentiating LMH from ERM foveoschisis.(15) The indication for surgery was based on a significant progressive decrease in distance VA, associated with a significant near VA impairment and an anatomical worsening.

### Surgical procedure

**Fig. 1:**
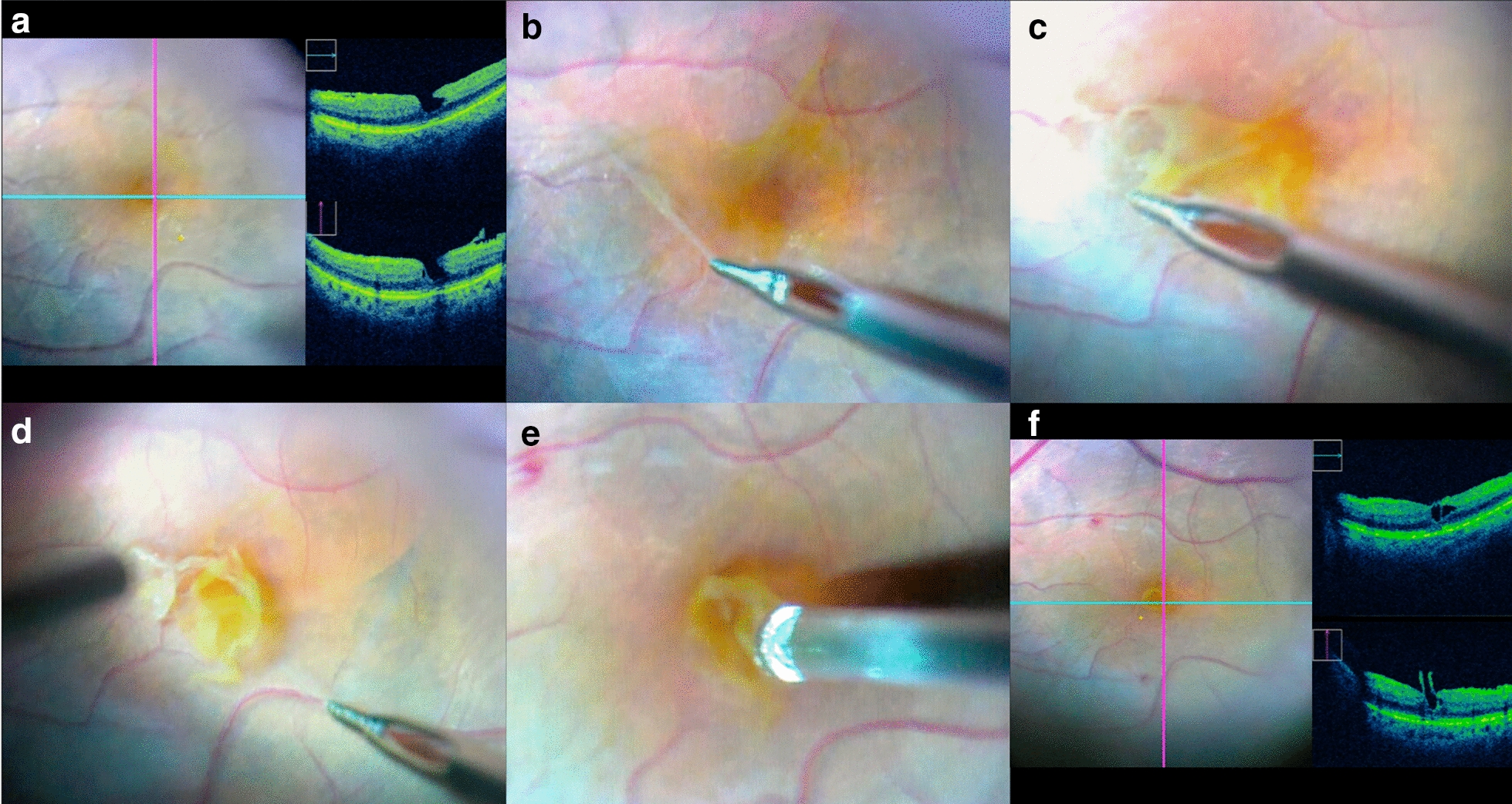
Intraoperative fundus pictures and optical coherence tomography (OCT) scans of a lamellar macular hole (LMH) with epiretinal proliferation. **a** Intraoperative OCT scan centered on the LMH after central vitrectomy and blue dye injection showing a partial retinal defect and a proliferative tissue. **b** Epiretinal proliferation appearing as a yellowish pigmented, thick and fluffy tissue. **c** This proliferation appeared strongly attached to the retina and seemed connected to the bottom of the hole, while it was centripetally peeled off towards the edges of the retinal defect. **d** The internal limiting membrane was removed after dual membrane blue dye staining. **e** Excess of proliferation was gently trimmed using the cutter probe. **f** At the end of the procedure, intraoperative OCT was used to assess the absence of full-thickness macular hole

All patients underwent 3-port 25-gauge PPV, under regional anesthesia with peribulbar block, following a common standardized procedure. A non-contact wide-angle viewing system (Resight, Carl Zeiss Meditec AG, Jena, Germany) or a contact lens (MiniQuad Vit; Volk, Mentor, OH) was used for wide-field visualization, and a flat contact lens (Phakos, Montreuil, France) for macular visualization. After core vitrectomy, posterior vitreous detachment was completed when necessary by applying suction with the vitrectomy probe around the optic nerve head and extended to the peripheral retina. Then, a mix of brilliant blue and trypan blue (Membrane Blue-Dual; DORC International, Zuidland, The Netherlands) was injected and left on the retinal surface for 1 min to stain the internal limiting membrane (ILM) and possible ERM. When present, an ERM was peeled off. In cases of LMH with epiretinal proliferation, the proliferative material was centripetally peeled off towards the fovea, but left attached to the edges of the hole. Excess of proliferation was trimmed using the vitrectomy probe when necessary (Fig. 1). In all cases, the ILM was peeled off around the hole, using a 25-gauge asymmetrical forceps (Alcon Grieshaber AG, Schaffhausen, Zuidland, Switzerland), while being careful not to pull on the epiretinal proliferation. In some cases, intraoperative SD-OCT images were obtained using the Rescan 700 device (Carl Zeiss Lumera 700 with integrative HD OCT, Carl Zeiss Meditec AG) to analyze changes in macular structure during the surgical steps, and to detect a potential full-thickness foveal opening at the end of surgery (Additional file 1: Video S1). Peripheral vitrectomy was then completed. A full air-fluid exchange and post-vitrectomy tamponade were performed at the surgeon’s discretion with either air or a mixture with 20% sulphur hexafluoride (SF6) or with 17% perfluoroethane (C2F6), without postoperative positioning. After removal of the trocars, sclerotomy incisions were sutured in case of leakage. A combined phaco-vitrectomy procedure was performed at the surgeon’s discretion.

Surgical details including the gauges used, completion of the posterior vitreoretinal detachment at the time of vitrectomy, peeling of an ERM, epiretinal proliferation and ILM, and the use of a tamponade agent were collected from the operative reports.

### Pre- and postoperative analysis

The main functional outcome was the change in distance VA between the preoperative examination and the last follow-up visit. The near VA and the presence of metamorphopsia were also compared.

The main anatomical outcome was the rate of foveal profile restoration, defined as a “LMH closure” 3 months post-surgery. Additionally, the foveal contour was subjectively defined as regular or irregular on postoperative SD-OCT, and the presence of a residual intraretinal cavitation was recorded. The presence of a disruption of the EZ and/or external limiting membrane (ELM), as well as the central foveal thickness (CFT) (defined as the mean value of the thinnest vertical distance between the bottom of the hole and the Bruch’s membrane, measured manually using the caliper on the high-definition central, vertical and horizontal line scans) were compared between the pre- and postoperative examinations. A disruption of the EZ and/or ELM was defined by a loss of continuity of these retinal layers within the central 1000-μm diameter circle of the ETDRS grid on the OCT B-scan.

Anatomical outcomes and complications were also assessed at the last follow-up examination on fundus examination and macular SD-OCT.

### Statistical analysis

Quantitative values are presented as a mean ± standard deviation (SD), while qualitative values are shown as a ratio and a percentage. The Wilcoxon’s signed-ranked test was used to compare pre- and postoperative VA and CFT. A *P* value less than 0.05 was considered statistically significant. All analyzes were performed using XLSTAT software (Assinsoft, Paris, France).

## Results

### Population

Eleven eyes of 11 patients were included in this study. Patient preoperative characteristics and surgical details are presented in Table 1. The mean time between the first diagnosis of LMH and the surgery was 25.5 ± 35.1 months (range: 4–120 months). The mean age at the time of surgery was 74.2 ± 8.2 years (range: 60.7–89.2 years), and the mean follow-up duration post-surgery was 7.2 ± 3.9 months (range: 3.4–12.8 months).

**Table 1:** Preoperative clinical data and surgical details

Patient No.	Sex	Age range	Follow-up (months)	ERM	Proliferation	Preop. lens status	Combined cataract surgery	ILM peeling	Tamponade agent
1	F	60.7	5	No	Yes	PCIOL	No	Yes	C2F6
2	F	66.4	7	No	Yes	Phakic	Yes	Yes	SF6
3	F	86.1	4	Yes	Yes	PCIOL	No	Yes	C2F6
4	M	76.7	4	Yes	No	Phakic	Yes	Yes	SF6
5	F	72.2	10	Yes	Yes	PCIOL	No	Yes	No
6	M	74.2	12	No	Yes	Phakic	Yes	Yes	SF6
7	M	75.4	4	Yes	Yes	Phakic	Yes	Yes	Air
8	M	72.9	4	No	Yes	PCIOL	No	Yes	C2F6
9	F	74.7	13	Yes	No	Phakic	No	Yes	SF6
10	F	67.3	12	No	Yes	PCIOL	No	Yes	SF6
11	M	89.2	4	Yes	Yes	PCIOL	No	Yes	No

ERM: epiretinal membrane; ILM: internal limiting membrane; PCIOL: posterior capsule intraocular lens

### Surgery

Posterior vitreous detachment was completed intraoperatively in 4 out of 11 cases (36.4%). In 9 cases (81.8%), epiretinal proliferation was present, and seen intraoperatively as a thick, dense, yellow material surrounding the hole and strongly adherent to the underlying retina (Fig. 1). Connections between the proliferation and the outer retinal layers within the hole were recorded in all cases. The ILM was easily removed in all cases. An extrafoveal ERM was present in 6 eyes (54.5%), and removed during ILM peeling. Five patients (45.5%) were phakic. A combined phaco–vitrectomy procedure was performed in 4 eyes (36.4%), and one eye (9.1%) secondarily underwent cataract surgery during the follow-up period. All patients were pseudophakic at the last examination.

### Functional outcomes

**Fig. 2:**
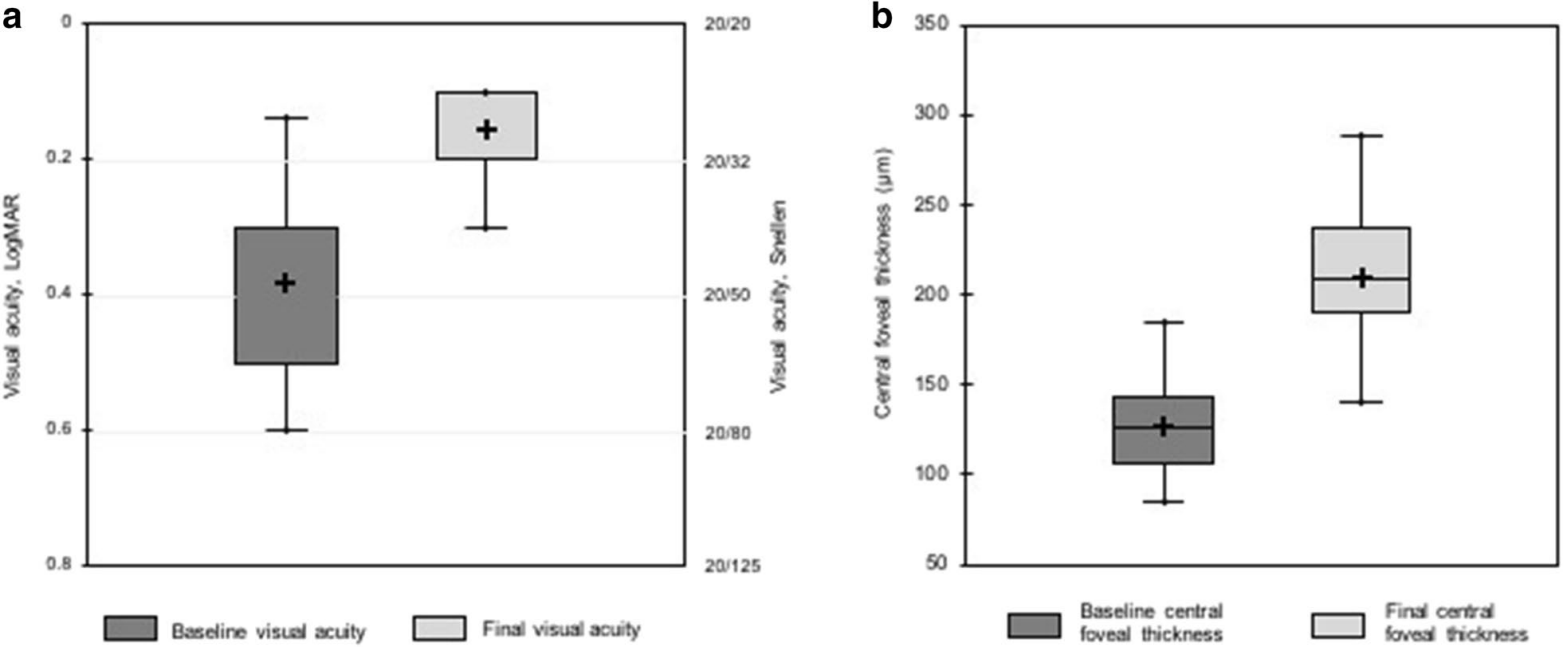
Preoperative and postoperative visual acuities (VA) and central foveal thickness (CFT). **a** VA significantly improved from 0.44 ± 0.19 LogMAR (20/55 Snellen equivalent) to 0.16 ± 0.08 LogMAR (20/28 Snellen equivalent), *P* = 0.02. **b** The mean CFT increased from 127.6 ± 29.2 μm (range: 85.5–185.0 μm) preoperatively to 209.0 ± 44.0 μm (range: 140.0–289.0 μm) postoperatively (*P* = 0.001)

The functional and anatomical outcomes of each individual patient are shown in Table 2. All patients experienced a functional improvement at the last follow-up visit. The VA significantly improved from 0.44 ± 0.19 LogMAR (20/55 Snellen equivalent; range: 0.80-0.20) to 0.16 ± 0.08 LogMAR (20/28 Snellen equivalent; range: 0.30-0.10; *P *=0.02) (Fig. 2a). The mean visual improvement between the preoperative examination and the last follow-up visit corresponded to 3 Snellen lines. The mean change in distance VA was not significantly different between eyes that underwent cataract surgery and eyes that did not (*P *= 0.81), and between eyes with and without associated extrafoveal ERM (*P *= 0.49) (Table 3). Five out of 8 patients with metamorphopsia (62.5%) experienced a resolution or a reduction of metamorphopsia, and the near VA improved in all patients (Table 2).

**Table 2:** Postoperative outcomes

Patient No.	Hole closure	Foveal profile	Retinal cavitation	Central foveal thickness (µm)		EZ disruption		ELM disruption		Distance VA (Snellen)		Near VA (Parinaud)		Metamorphopsia	
				Preop	Final	Preop	Final	Preop	Final	Preop	Final	Preop	Final	Preop	Final
1	Yes	Regular	Yes	96	208	Yes	No	Yes	No	20/80	20/25	8	4	Yes	No
2	Yes	Irregular	Yes	185	218	No	No	No	No	20/50	20/25	4	3	Yes	Yes
3	Yes	Irregular	No	126	289	Yes	No	Yes	No	20/63	20/32	6	3	Yes	No
4	Yes	Regular	No	153	238	Yes	No	Yes	No	20/125	20/40	4	3	No	No
5	Yes	Irregular	No	116	140	Yes	No	Yes	No	20/63	20/32	4	2	Yes	No
6	Yes	Regular	No	86	199	Yes	No	No	No	20/63	20/25	5	2	Yes	Yes
7	Yes	Irregular	Yes	143	242	No	No	No	No	20/63	20/40	3	3	Yes	No
8	Yes	Regular	No	126	234	Yes	Yes	Yes	Yes	20/50	20/25	5	3	No	No
9	Yes	Regular	No	141	207	Yes	Yes	No	No	20/32	20/25	4	3	No	No
10	Yes	Regular	No	144	182	Yes	Yes	Yes	Yes	20/32	20/25	4	2	Yes	Yes
11	Yes	Regular	No	90	141	Yes	No	Yes	No	20/32	20/25	4	2	Yes	No

EZ: ellipsoid zone; ELM: external limiting membrane; VA: best-corrected distance visual acuity

**Table 3:** Pre- and postoperative visual acuity changes according to cataract surgery, and the presence of associated epiretinal membrane

	Preoperative VA LogMAR, mean ± SD (Snellen equivalent)	Last follow-up VA LogMAR, mean ± SD (Snellen equivalent)	VA gain LogMAR, mean ± SD	*P* value
Cataract surgery				0.81
With cataract surgery (n = 5)	0.48 ± 0.22 (20/60)	0.18 ± 0.11 (20/30)	0.30 ± 0.15	
Without cataract surgery (n = 6)	0.40 ± 0.17 (20/50)	0.13 ± 0.05 (20/26)	0.27 ± 0.15	
Epiretinal membrane				0.49
With ERM (n = 5)	0.50 ± 0.23 (20/63)	0.20 ± 0.09 (20/31)	0.25 ± 0.15	
Without ERM (n = 6)	0.40 ± 0.15 (20/50)	0.10 ± 0.00 (20/25)	0.32 ± 0.15	

VA: Visual acuity; LogMAR: logarithm of the minimum angle of resolution; SD: standard deviation; ERM: epiretinal membrane

### Anatomical outcomes

**Fig. 3:**
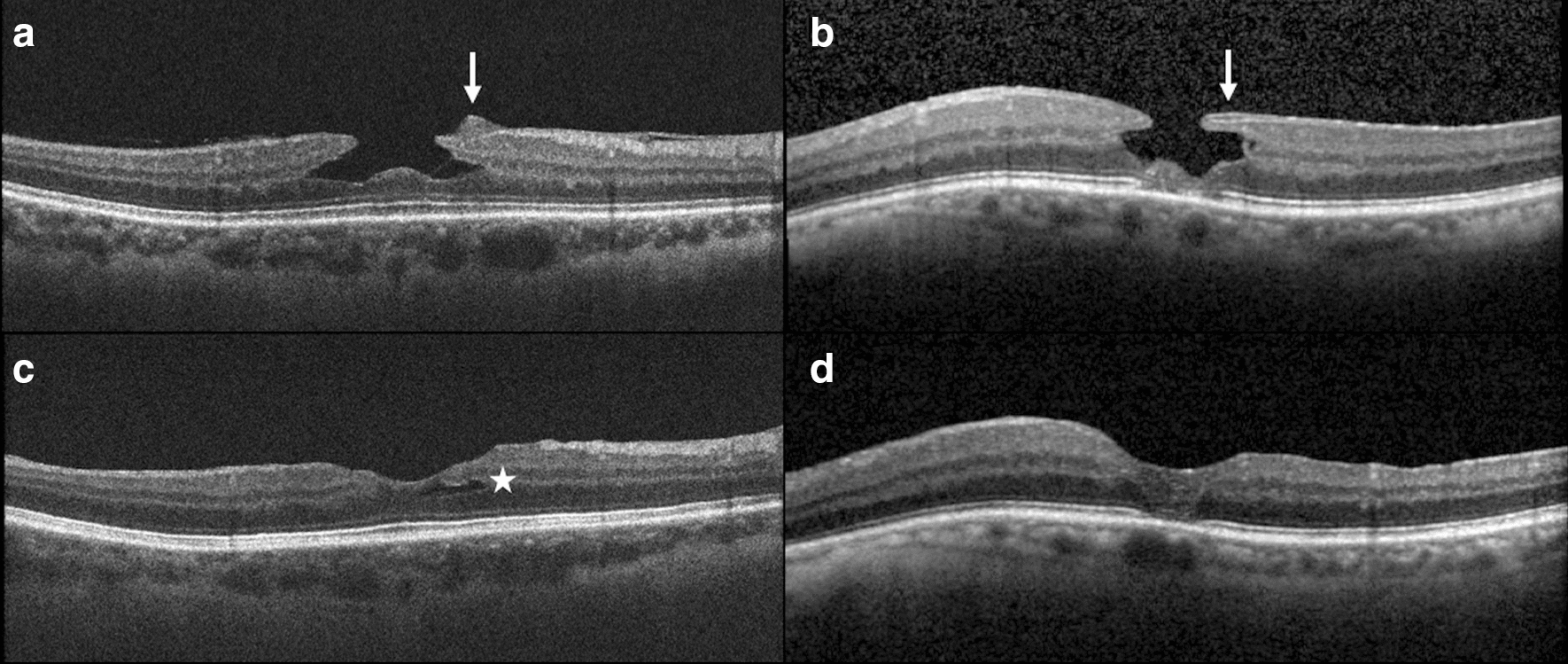
Preoperative and postoperative spectral-domain optical coherence tomography (SD-OCT) scans. **a**, **b** Preoperative SD-OCT scans showing two cases of lamellar macular hole (LMH). Epiretinal proliferation is visible as an additional tissue of medium reflectivity at the edge of the hole (white arrows). **c**, **d** Postoperative SD-OCT scans showed a restoration of the foveal contour. In the first presented cases (**c**), an intraretinal residual cavitation was present postoperatively (white star)

Three months post-surgery, all eyes (11/11 eyes) showed a restoration of the foveal profile on SD-OCT, corresponding to a LMH closure (Table 2 and Fig. 3). Seven eyes (63.6%) showed a regular foveal contour, while 4 eyes (36.4%) had an improved but still irregular foveal profile. Remaining intraretinal cavitations were seen in 3 eyes (27.3%). Postoperative en face OCT images showed typical hyporeflective defects of the macular surface resulting from the dissociation of the optic nerve fiber layer in the area of ILM peeling.

On the preoperative OCT scans, 9 eyes (81.8%) showed EZ disruption and 7 eyes (63.6%) had both ELM and EZ disruption on at least one OCT scan passing through the fovea. Three months post-surgery, 6 out of the 9 eyes (66.7%) showed a restoration of the EZ, and 5 out of the 7 eyes (71.4%) showed a restoration of the ELM. In the 5 eyes with restored ELM, the EZ was also restored. No eyes experienced EZ or ELM disruption after surgery. The CFT increased in all cases, and the mean CFT increased significantly from 127.6 ± 29.2 μm (range: 85.5–185.0 μm) preoperatively to 209.0 ± 44.0 μm (range: 140.0-289.0 μm) postoperatively (*P* = 0.001) (Fig. 2b), with a mean CFT gain of 81.4 ± 43.1 μm (range: 24.0–163.5 μm).

### Complications

No cases of FTMH were reported. One patient experienced postoperative vitreous hemorrhage with spontaneous resolution within 2 weeks.

## Discussion

In this study, all included patients, carefully selected based on recent OCT diagnostic criteria, experienced foveal profile restoration and a VA gain after vitrectomy with epiretinal proliferation peri-hole peeling [2].

Previous studies on the natural course of LMH have shown that LMH are generally anatomically and functionally stable [16, 17]. However, some patients may experience progressive anatomical changes, including a disruption of the outer retinal layers, leading to functional impairment and raising questions about the benefit of surgery [7].

Visual and anatomical outcomes after vitrectomy for LMH vary widely between the studies published in recent years [11, 18–22]. This discrepancy could be explained by the different surgical approaches used, but also by a misclassification of other macular conditions that differ from LMH such as macular pseudoholes and ERM foveoschisis. Using en face OCT, our team has previously distinguished macular pseudoholes from LMH, and also demonstrated that eccentric epicenters of ERM may correspond to a pseudohole with intraretinal cleavage (i.e. ERM foveoschisis) [15].

The pathogenesis of LMH remains largely unknown, but one hypothesis is that it could occur after a posterior vitreous detachment as an abortive process of FTMH formation [15, 23]. Consequently, LMH eyes may present a concomitant ERM, but the tractional effect of the ERM does not play a major role in the genesis of a “true” LMH, which should therefore be distinguished from other tractional diseases (i.e. macular pseudoholes and ERM foveoschisis) [2, 14, 15]. This finding has recently been stressed by Hubschman and co-workers, who have provided a clear-cut OCT-based definition of LMH, macular pseudoholes and ERM foveoschisis [2]. This distinction is not only semantic, but has also significant clinical consequences. Indeed, while there is no doubt about the benefit of ERM peeling for macular pseudoholes and ERM foveoschisis, LMH surgery remains controversial with inconsistent outcomes [3, 4, 6, 15, 24]. Prior LMH studies based on the earlier classification could have included cases of MPH and ERM foveoschisis, and could therefore have reported better surgical outcomes than usually found, while using conventional macular peeling approaches [19, 21–23, 25].

Consequently, the cases included in the present report were carefully selected using OCT B-scans and en face OCT, and eyes with ERM were included only in case of extra-foveal location without tangential foveal traction on the en face OCT image. Recent OCT studies have shown that 50–75% of LMH are associated with a non-tractional epiretinal proliferation [7, 17, 26]. On direct visualization, this proliferative material had a yellowish appearance, and no yellow pigment has been reported within the retina other than the xanthophyll pigment, which is located within the Henle fiber layer. Histologically and immunohistochemically, glial cells and in particular Müller cells have been found in this proliferative material, supporting the assumption that it could originate from the middle retinal layers and that its development could be triggered by the presence of an inner retinal break in LMH eyes [27]. These findings could explain the high occurrence rate, up to 50%, of post-operative FTMH in previous series on LMH surgery [4–6, 25]. Indeed, we assumed that, in these cases, FTMH formation was secondary to a rupture of the outer retinal layers by direct traction on this proliferative tissue.

To reduce this risk, epiretinal proliferation was centripetally peeled off, but left attached to the edges of the holes in this study in order not to impair its connections with the underlying retinal layers, and this could explain the absence of postoperative FTMH. In line with our results, other groups have recently shown positive outcomes using different variants of this technique, including a double inverted epiretinal proliferation and ILM flap technique [8–10]. Due to the small number of patients included in these recent studies, the best surgical approach to be used remains to be determined. However, these papers as well as ours suggest that the use of a modified macular peeling technique, limiting the traction on the epiretinal proliferation and residual foveal tissue, based on peri-hole peeling of the proliferative tissue or the use of an inverted or double inverted flap technique, could improve the surgical outcomes of patients with LMH.

While in a normal retina, the ILM separates the retinal tissue from the vitreous cavity, in LMH eyes the vitreous cells are in direct contact with the retina. Macrophage-like cells from the vitreous could therefore infiltrate the retina, and stimulate retinal gliosis, leading to the development of epiretinal proliferation, or alternatively to the progressive loss of the retinal tissue [28–31]. The regulatory factors involved, such as tissue necrosis factor-α, have not yet been studied in LMH, and further proteomic analyzes are needed to confirm this hypothesis and to improve our understanding. Vitrectomy with modified macular peeling surgery could allow interrupting this degenerative process, and help to restore the retinal homeostasis by restoring the foveal architecture. Additionally, the use of epiretinal proliferation and/or ILM to fill the retinal defect could provide a scaffold for Müller cell proliferation with a potential support for the photoreceptors. Given that epiretinal proliferation is supposed to originate from the retina and to contain Müller glial cells, it could, when left over the hole at the end of the peeling, provide glial Müller cells that could have the potential to regenerate all retinal cells in animal model studies [32, 33]. Therefore, the use of epiretinal proliferation to fill the retinal defect may be responsible for the restoration of the foveal contour and the improvement of EZ and ELM disruption (71.4% and 66.7% of cases, respectively), with subsequent VA improvement.

The need for ILM peeling and gas tamponade in LMH surgery remains questionable with no evidence of its benefit in LMH surgery [34, 35]. In our series, the ILM peeling was however performed in all cases to relax the retina and to potentially contribute to the restoration of the foveal contour. Due to the lack of consensus on the surgical indication and technique to be used for LMH, we performed vitrectomy with gas tamponade in most cases, first to potentially improve the likelihood of hole closure by retaining the proliferation within the hole, and second to potentially prevent FTMH evolution. Two patients were operated without tamponade and they both achieved similar good results. In these two cases, the decision was taken intraoperatively, based on the intraoperative OCT images [36]. Increased performance of the current surgical systems, including the relatively recent use of intraoperative OCT, could have contributed to a better intraoperative visualization of the details of the retina, and could therefore have contributed to a safer surgical approach limiting the traction on the epiretinal proliferation.

The limitations of this study include its small sample size and its retrospective design. Due to the lack of consensus on LMH surgery and the scarcity of patients with typical LMH complaining about a significant progressive loss of vision, a small number of patients were included. Four patients underwent combined phaco-vitrectomy and another one underwent cataract surgery during the follow-up period. Cataract surgery could have contributed to the functional outcomes. However, the patients who underwent vitrectomy alone showed a similar visual recovery, the near VA improved in all patients and 5 out of the 8 cases with metamorphopsia reported a resolution of metamorphopsia. The strengths of this study are the well-defined study population that was selected based on restrictive criteria using detailed SD-OCT, and the use of standardized protocols for surgical procedures and follow-up examinations.

To conclude, this study showed that surgery could be a therapeutic option when patients with a “true” LMH experience a worsening of the visual function and anatomical OCT features. Cautious and non-traumatic macular peeling surgery could restore the foveal architecture, allowing restoring retinal homeostasis and interrupting the degenerative process. However, surgery for LMH remains indicated on a case-by-case basis. Further large multicentric studies are needed to confirm our assumptions and to define more precisely the best surgical approach to be used and the functional and anatomical criteria for surgery.

## **Supplementary Information**

**Additional file 1.** Supplemental digital content. Surgical video of case of a lamellar macular hole with epiretinal proliferation. After a core vitrectomy, membrane blue dual dye was injected to stain the internal limiting membrane. The proliferative tissue was centripetally peeled off toward the edges of the retinal defect and the internal limiting membrane peeled off around the fovea. After the macular peeling, excess of proliferation was trimmed using the cutter probe. Intraoperative OCT was used to assess the absence of full-thickness macular hole at the end of the procedure and no tamponade agent was used in this case.

## Data Availability

All deidentified and coded data of patients included in the study are available by request.

## Abbreviations

CFT: Central foveal thickness
ELM: External limiting membrane
ERM: Epiretinal membrane
EZ: Ellipsoid zone
FTMH: Full-thickness macular hole
ILM: Internal limiting membrane
LHEP: Lamellar hole-associated epiretinal proliferation
LMH: Lamellar macular hole
PPV: Pars plana vitrectomy
SD: Standard deviation
SD-OCT: Spectral-domain optical coherence tomography
VA: Visual acuity
